# Resting-state dopaminergic cell firing in the ventral tegmental area negatively regulates affiliative social interactions in a developmental animal model of schizophrenia

**DOI:** 10.1038/s41398-021-01346-2

**Published:** 2021-04-22

**Authors:** Hidekazu Sotoyama, Hisaaki Namba, Yutaro Kobayashi, Taku Hasegawa, Dai Watanabe, Ena Nakatsukasa, Kenji Sakimura, Tomoyuki Furuyashiki, Hiroyuki Nawa

**Affiliations:** 1 Department of Molecular Neurobiology, Brain Research Institute, Niigata University, Niigata, 951-8585 Japan; 2 Department of Physiological Sciences, School of Pharmaceutical Sciences, Wakayama Medical University, Wakayama, 640-8156 Japan; 3 Department of Biological Sciences, Graduate School of Medicine, Kyoto University, Kyoto, 606-8501 Japan; 4 Department of Animal Model Development, Brain Research Institute, Niigata University, Niigata, 951-8585 Japan; 5 Division of Pharmacology, Graduate School of Medicine, Kobe University, Hyogo, 650-0017 Japan

**Keywords:** Molecular neuroscience, Schizophrenia

## Abstract

Hyperdopaminergic activities are often linked to positive symptoms of schizophrenia, but their neuropathological implications on negative symptoms are rather controversial among reports. Here, we explored the regulatory role of the resting state-neural activity of dopaminergic neurons in the ventral tegmental area (VTA) on social interaction using a developmental rat model for schizophrenia. We prepared the model by administering an ammonitic cytokine, epidermal growth factor (EGF), to rat pups, which later exhibit the deficits of social interaction as monitored with same-gender affiliative sniffing. In vivo single-unit recording and microdialysis revealed that the baseline firing frequency of and dopamine release from VTA dopaminergic neurons were chronically increased in EGF model rats, and their social interaction was concomitantly reduced. Subchronic treatment with risperidone ameliorated both the social interaction deficits and higher frequency of dopaminergic cell firing in this model. Sustained suppression of hyperdopaminergic cell firing in EGF model rats by DREADD chemogenetic intervention restored the event-triggered dopamine release and their social behaviors. These observations suggest that the higher resting-state activity of VTA dopaminergic neurons is responsible for the reduced social interaction of this schizophrenia model.

## Introduction

The impairment of human social behavior is one of the major pathological traits of psychiatric disorders such as schizophrenia and depression^1–3^. The neuropharmacology of antipsychotics or antidepressants medicating these social deficits indicates that monoamines like dopamine and serotonin are involved in the neuropathology of social behaviors in these disorders^4–7^. These monoamines are known to independently and/or interactively regulate animal social behaviors through cognitive and/or instinctive processes^8–11^. The fact that the treatment effects of antipsychotics and antidepressants only emerge after their long-term intake suggests a pathological role of chronic dysregulation of monoaminergic neurotransmission in these disorders^12–15^. However, the pathophysiological nature of chronic monoaminergic dysfunctions underlying social deficits remains to be elucidated in schizophrenia^16,17^.

Dysfunction of the brain dopaminergic system is implicated in the pathophysiology of schizophrenia, in particular its positive symptoms^17,18^. Previous brain imaging studies of schizophrenia patients suggest that hyperdopaminergic states in their nigrostriatal pathway (i.e., A9 pathway), as well as hypodopaminergic states in their mesocorticolimbic pathway (i.e., A10 pathway), are associated with the positive, cognitive, and potentially negative symptoms of schizophrenia^19–22^, although many controversies remain^23,24^. It is sometimes unclear whether the reported dopaminergic states of patients reflect a baseline resting condition, an acute event-related response, or a mixture of both^25–27^. In addition, it is also arguable whether the task/test-related dopamine release encodes a predictive function for the task or reflects just a neural response to the task^28,29^. Therefore, the real nature of the patients’ dopaminergic dysfunction in their social deficits remains to be clarified. Which has a crucial role in the regulation of social behaviors, baseline- or event-triggered dopamine release^26,30,31^?

We attempt to demonstrate the validity of the obstetric complication hypothesis of schizophrenia, using various animal models mimicking the inflammatory conditions of obstetric complication^32–34^. Among those, we have been intensively exploring the animal model that is established by perinatal exposure to an amniotic cytokine, epidermal growth factor (EGF). Rodents in this model develop post-pubertal hyperdopaminergic states with several behavioral deficits such as decreased social behaviors, reduced prepulse inhibition, elevated latent inhibition of fear learning, etc.^35–39^, most of which are presumably associated with positive symptoms of schizophrenia. However, it remains to be investigated how the hyperdopaminergic state of this model is associated with the behavioral endophenotype relevant to the negative symptoms of schizophrenia^40^. In the present study, therefore, we explored the regulatory role of post-pubertal hyperdopaminergic states in social interaction deficits, employing in vivo single-unit recording and in vivo microdialysis of dopaminergic neurons in the ventral tegmental area (VTA). We also manipulated the dopaminergic cell firing at resting state using designer receptors exclusively activated by designer drugs (DREADD)^41^ and attempted to verify the role of resting-state dopaminergic states in the regulation of same-gender affiliative social interaction in this EGF model.

## Materials and methods

### Animals

We purchased male Sprague-Dawley rats (postnatal day 2) with their dams from SLC (Hamamatsu, Shizuoka, Japan). Male Long-Evans rats (postnatal week 6–8) were obtained from Charles River Japan (Yokohama, Japan). The Cre-driver rats (Long Evans-Tg(TH-Cre)3.1Deis) were provided by Rat Resources and Research Center (University of Missouri, Columbia, Missouri USA)^42^. Rats were housed in polypropylene cages (58 cm L × 28 cm W × 24 cm H) and given free access to food and water. Rats were grown in a temperature-controlled room (22.0 ± 1.0 °C) and maintained under a 12-h light-dark cycle (8:00 on and 20:00 off). At postnatal weeks 10–16, we carried out the following experiments. Cre-driver rats were subjected to the test sequence of social interaction, in vivo microdialysis, and single-unit recording under anesthesia. Some rats were dissected during the experimental sequence and utilized for immunoblotting or immunohistochemistry (Supplemental Table S1). We excluded the data of the rats that lost a dialysis probe or an implanted electrode. All animal experiments were performed at the day cycle, approved by the Animal Use and Care Committee of Niigata University, and performed in accordance with the Guiding Principles for the Care and Use of Laboratory Animals (National Institutes of Health, Bethesda, MD, USA). All efforts were made to minimize both the suffering and the number of animals used in this study. Randomization of animal allocation was not applied in the following experimental groups. The investigators were blinded only for the manual counting of sniffing duration in the social interaction test.

### AAV vector injection

We obtained AAV5 viral vectors carrying genes for hM4Di and mCherry (AAV5-hSyn-DIO-hM4DiGi-mCherry) or mCherry alone (AAV5-hSyn-DIO-mCherry) from Addgene (Watertown, MA, USA). Rats were anesthetized with the mixture of midazolam (2.0 mg/kg; Dormicum; Astellas Pharma Inc., Tokyo, Japan), medetomidine (0.38 mg/kg; Domitol; Nippon Zenyaku Kogyo. Co., Fukushima, Japan), and butorphanol (2.5 mg/kg; Vetorphale, Meiji Seika Pharma Co., Tokyo, Japan). We microinjected one microliter of the vector solution (5.2–6.1 × 10^12^ vg/mL) to both hemispheres of VTA of Cre-driver rats (bregma in mm: AP −5.2, ML ±0.5, DV − 7.3) at the speed of 0.2 µL/min.

### Drug treatment

The EGF rat model was prepared as described previously^43^. In brief, recombinant human EGF (0.875 mg/kg; Higeta Shoyu Co., Chiba, Japan) was administered subcutaneously to half of each litter daily from postnatal day 2–10. EGF-treated rats (referred to EGF model rats hereafter) and saline-treated rats (referred to control rats hereafter) were grown to the post-pubertal stage (postnatal week 10–12), and some of those animals further received risperidone (1.0 mg/kg/day, i.p.; Rispadal®; Janssen Pharmaceuticals Inc., Tokyo, Japan) for 10–14 days prior to the following behavioral and/or electrophysiological tests. Clozapine N-oxide (CNO; Hello Bio Ltd., Bristol, UK) solution (50 mg/mL dimethyl sulfoxide) was loaded into an osmotic pump (Alzet Type2002; Alzet, DURECT Corp., Cupertino, CA, US). The pump was inserted into the space between the skin and spinal muscle, supplying CNO at a rate of 600 μg/day/rat (~1.5 mg/kg/day) for 10–13 days prior to the following behavioral and/or electrophysiological tests.

### In vivo single-unit recordings

Extracellular single-unit recordings from freely moving rats were performed with the aid of a wireless head amplifier system linked to a Bluetooth transmitter/receiver, which was originally developed by Hasegawa et al.^44^. The microwire array probe was prepared with modifications according to a previous report^45^; it consisted of a bundle of four shield tungsten wires (50 µm diameter; California Fine Wire, Grover Beach, CA, USA) extending 5 mm beyond the tip of a glass guide cannula (TSP100170; Neuralyux, Bozeman, MT, USA). One wire served as the reference. The probe was positioned at the VTA by monitoring unit discharges as described above. Spike shapes and frequencies were analyzed in time by customized Labview software^44^, and unit activities that matched the criteria described below were stored and analyzed off-line using the Spike 2 software (Cambridge Electric Design, Cambridge, UK). The mean firing rate and spikes within bursts (SWB) of each unit were calculated according to the established criteria^46^.

Extracellular single-unit recordings were also performed under chloral hydrate anesthesia (400 mg/kg, i.p.)^47,48^. Anesthetized rats were mounted on a stereotaxic apparatus, and their body temperature was continuously kept at 37.0 ± 0.5 °C. A glass microelectrode filled with 0.5 M NaCl (resistance; 8–20 MΩ) was inserted into the anterior region of VTA (all bregma in mm: AP −4.8 to −5.4, ML 0.6 to 1.0, DV −7.0 to −8.5), the locus coeruleus (LC) (AP −11.2 to −11.3 with a +15° angle toward the posterior direction, ML 1.1 to 1.4, DV −4.8 to −6.5), or the dorsal raphe (AP −7.5 to −8.0 with a +10° angle toward the lateral direction, ML 8.0 to 1.2, DV −4.8 to −6.1). Neuronal signals were recorded for 2–5 min and amplified using an Axoclamp 2B (Molecular Devices, CA, USA) connected to a high gain amplifier (AVH-11; Nihon Kohden, Tokyo, Japan). Single units were constantly monitored using the window discriminator (121 Window Discriminator; World Precision Instruments, FL, USA). The signals were transferred via a digitizer (Digidata 1200; Molecular Devices) to a computer equipped with recording software (Axoscope 1.1; Molecular Devices).

We identified the activities of putative dopaminergic neurons in the VTA by their characteristic profiles: (1) the typical triphasic action potential with a marked negative deflection, (2) the characteristic long duration (>2.0 ms) with the action potential width from start to the negative trough >1.1 ms, and (3) the slow firing rate (<10 Hz) with an irregular single spiking pattern and occasional bursting activity^48^. We distinguished the activities of putative serotonergic neurons in the dorsal raphe as described previously^49^: (1) the typical biphasic action potential with a marked negative deflection, (2) the characteristic duration (0.2–4.0 ms) with the action potential width from start to the negative trough >1.1 ms, and (3) a regular rhythmic spiking pattern of activity. The activities of putative noradrenergic neurons in the LC were isolated based on the following characteristic waveform and their sensory response^50,51^: (1) the typical triphasic action potential with rather longer duration (action potential width from onset to the negative trough >1.1 ms), (2) the regular rhythmic and slow firing rate (<6 Hz), and (3) the biphasic excitation-inhibition response to pinching of the contralateral hind paw.

### In vivo microdialysis

Rats were anesthetized with the same anesthetic mixture and subjected to in vivo microdialysis as described by Sotoyama et al.^39^. In brief, the stereotaxic coordinates were targeted to the boundary of the prelimbic-infralimbic cortex (bregma in mm: AP +3.2, ML 0.8, DV −4.7). In rats having received the AAV injection, the probe implantation was done 24–30 days after the injection. Microdialysis experiments were performed after allowing the rat at least 10 days of recovery from surgery. The microdialysis probe (3 mm active area; A-I-8-03; Eicom Ltd, Kyoto, Japan) was perfused with artificial cerebrospinal fluid^39^ (pH 7.0) at a flow rate of 2.0 µL/min. Alternatively, the flow rate was reduced to 0.7 µL/min to increase baseline dopamine concentrations in the resting condition. Dopamine concentrations in the dialysates were determined as reported previously^39^. Data were not compensated for the recovery rate.

### Social interaction

The index for same-gender affiliative social interaction was measured in the modestly aversive condition^52^. In brief, an adult male rat was first subjected to the exploratory locomotion test in an open field chamber (45 cm L × 45 cm W × 30 cm H, MED Associates, St. Albans, VA, USA) for 60 min. After acclimatization with the test condition, the rat was exposed to an age-, bodyweight-, and sex-matched unfamiliar control rat in the same chamber under a moderate light level (400 Lx) for 10 min. All testing was videotaped and scored in a blinded fashion with the aid of the video tracking software EthoVision XT (Noldus; Wageningen, the Netherlands). Scoring of social interaction duration by experimentors was based on sniffing behaviors, defined as active chasing of the partner, shaking the nose near the partner, and contacting the partner with the nose.

### Statistical analysis

Results were expressed as means ± SEM. All data were subjected to Box’s M test or Levene’s test for between-group homogeneity of their variances and Kolmogorov–Smirnov test or Shapiro–Wilk test for their fitting to the Gaussian distribution. The statistical justification and values for each method is shown in Supplemental Table S2. The sample sizes of animals for electrophysiological recording and behavioral tests were adjusted according to previous authentic studies^43–52^. One-dimensional data from multiple groups were subjected to analysis of variance (ANOVA) or Kruskal–Wallis test, followed by Tukey or Steel post hoc test, respectively. When data were obtained from only two groups, Mann–Whitney *U* test or two-sided Student’s/Welch’s *t*-test was used. We reduced the dimension of time or rat group, and employed their means to estimate the main effect of rat group or the event. The compressed data were subjected to any of the above statistical methods with the Holm’s compensation for their multiple comparisons. If the main effect of rat group and event was estimated to be significant, post hoc tests were performed at each time bin. When we used Mann–Whitney *U* test at each time bin, however, the analyses were not subjected to the Holm’s compensation to avoid type 2 errors. The alpha level was fixed to be 0.05. The Holm’s compensation was reflected by multiplying *P* value with Holm’s N. Statistical analyses were performed using the software of BellCurve for Excel (SSRI com. Tokyo, Japan).

## Results

### Increased VTA dopaminergic cell firing in the EGF model for schizophrenia

**Fig. 1:**
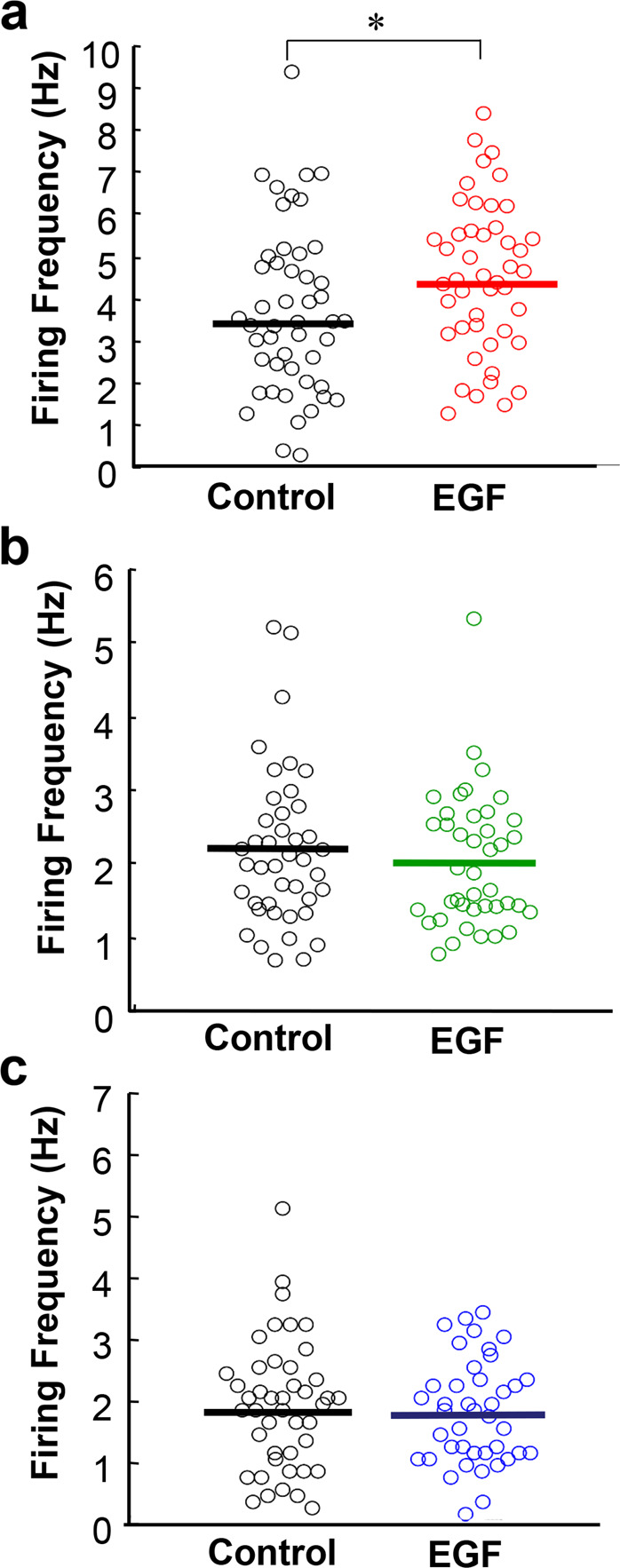
Differences in spontaneous neural activities of putative monoaminergic neurons in epidermal growth factor model rats. In vivo extracellular single‐unit recordings in the ventral tegmental area (VTA), locus coeruleus (LC), and dorsal raphe nucleus (DRN) of anesthetized rats. **a** The distributions of firing rates of putative VTA dopaminergic neurons are compared between the EGF model and control rats (*n* = 48 cells from 5 control rats, *n* = 46 cells from 5 EGF model rats,). **b** The distributions of firing rates of putative LC noradrenergic neurons are similar (*n* = 42 cells from 7 control rats, *n* = 41 cells from 7 EGF model rats,). **c** The distribution of firing rates of putative DRN serotonergic neurons are comparable *n* = 44 cells from 5 control rats, *n* = 41 cells from 5 EGF model rats). *P < 0.05, Mann–Whitney *U* test.

Dopamine, serotonin, and noradrenaline have all been implicated in the regulation of social behavioral traits^5,6^. To estimate the relative effects of individual monoaminergic cell activities on social behaviors, extracellular spike activities of three monoaminergic cell populations were determined in EGF model rats, which are known to show social interaction deficits^35^. We identified putative dopaminergic, serotonergic, and noradrenergic cells in the VTA, dorsal raphe nucleus (DRN), and LC, respectively, based on their reported particular firing properties and sensory responses (see details in the Materials and methods section). We measured their spontaneous firing frequencies under anesthetic conditions and compared those between control and EGF model rats (Fig. 1).

The frequency of putative dopaminergic cells in the VTA was significantly different between the EGF model and control rats (*U* = 811, *P* = 0.027; 3.59 ± 0.28 Hz in control rats and 4.41 ± 0.26 Hz in EGF model rats (Fig. 1a). However, the burst index SWB of EGF model rats (19.1 ± 2.6%) did not significantly differ from that of control rats (17.2 ± 2.9%) (*U* = 1104, *P* = 0.227). The frequency of putative noradrenergic cells in the LC was indistinguishable between groups (*U* = 805.5, *P* = 0.616); 2.25 ± 0.17 Hz in control rats and 2.09 ± 0.15 Hz in EGF model rats (Fig. 1b). Similarly, we failed to detect a difference in the firing frequency of putative serotonergic neurons in the DRN (*U* = 866.5, *P* = 0.754); 1.88 ± 0.16 Hz in control rats and 1.76 ± 0.13 Hz in EGF model rats (Fig. 1c). Among the three monoaminergic activities, only the spiking frequency of putative VTA dopaminergic cells reflected the abnormality of EGF model rats under the present experimental condition, although the authenticity of this cell criterion of the serotonergic neurons remains to be controversial^53^.

### Elevated dopaminergic activity of EGF model rats under freely moving conditions

**Fig. 2:**
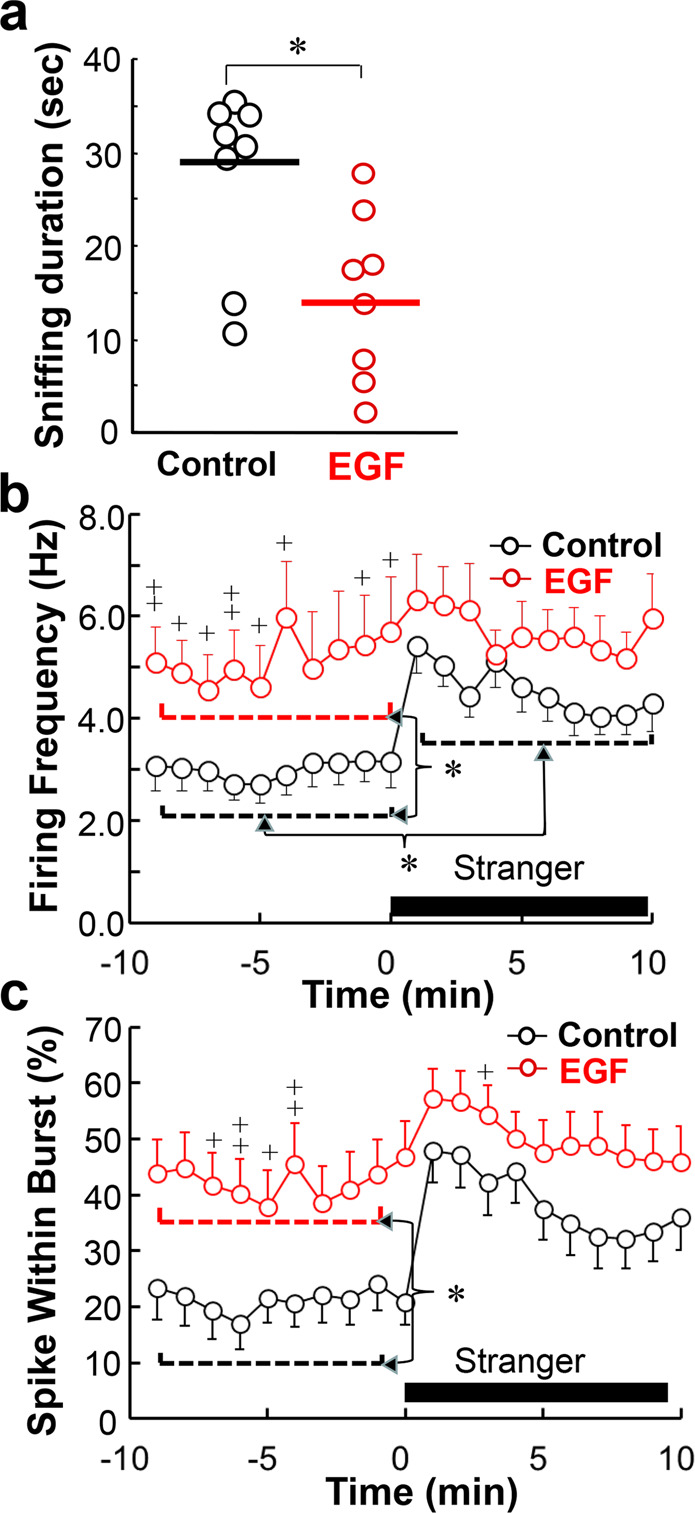
Spiking activities of putative dopaminergic neurons in the ventral tegmental area during social interaction tests. **a** Social interaction scores in epidermal growth factor (EGF) model rats (*n* = 8) and control rats (*n* = 8) were assessed by same-gender affiliative sniffing under mild aversive conditions (see details in the “Materials and methods” section) and compared by Mann–Whitney *U* test. **b** In vivo extracellular single‐unit recording in the VTA of freely moving rats with the aid of a wireless system. Putative dopaminergic cell firing was isolated from control rats (20 cells/12 rats and EGF model rats (17 cells/13 rats) and monitored during the social interaction with an unfamiliar male rat (Stranger). Averaged overall frequency of each rat group before and after social stimulation was calculated to estimate the main effects and then compared by Mann–Whitney *U* test with the Holm’s compensation; ^*^P < 0.05. In addition, the mean firing frequency during each 1 min time bin was compared between rat groups by Mann–Whitney *U* test without the Holm’s compensation; ^+^*P* < 0.05, ^++^*P* < 0.01. **c** Mean spike within bursts (SWB, %) was calculated from the same single‐unit recording. Averaged overall SWB of each rat group before and after social stimulation was similarly calculated and compared to each other by two-way ANOVA followed by Tukey post hoc test. In addition, mean SWB at each time bin was compared between rat groups by Mann–Whitney *U* test without the Holm’s compensation; ^+^*P* < 0.05, ^++^*P* < 0.01.

Futamura et al.^35^ initially reported decreased sniffing in EGF model rats under modestly aversive test conditions. In the present study, we first attempted to replicate these data (Fig. 2a). The sniffing duration of EGF model rats (14.4 ± 3.1 s/10 min) was reduced to half of that of control rats (27.7 ± 3.4 s/10 min). We monitored the single-cell unit activity of putative VTA dopaminergic neurons under the free-moving condition of the social interaction paradigm (Fig. 2b, c). During the resting state from −10 min to 0 min, the mean baseline spike frequency of putative dopaminergic cells was markedly higher in EGF model rats (5.16 ± 0.84 Hz) than in control rats (3.00 ± 0.36 Hz; *U* = 82, *P* = 0.021 with Holm’s compensation) (Fig. 2b). After the social interaction was initiated, however, there was no difference in spike frequency between groups (4.55 ± 0.40 s for control rats, 5.71 ± 0.62 s for EGF model rats, U = 115, *P* = 0.118 with Holm’s compensation). The significant difference in firing frequency between pre- and post-social test was found only in control rats (*U* = 104, *P* = 0.027 with Holm’s compensation) but not in EGF model rats (*U* = 29, *P* = 0.459 with Holm’s compensation).

A similar tread was observed in the measure of dopaminergic cell bursting, SWB (Fig. 2c). Two-way ANOVA for mean SWB revealed significant main effects of EGF challenge (*F*_1,70_ = 10.1, *P* = 0.002) and social stimuli (*F*_1,70_ = 6.11, *P* = 0.016) without an EGF × social interaction (*F*_1,70_ = 0.91, *P* = 0.343). Post hoc tests detected SWB differences between rat groups only in the baseline frequency measured at the resting state (21.1 ± 3.9% for control rats, 42.4 ± 6.0% for control rats, *P* = 0.024, by Tukey post hoc). These results indicate neurophysiological deficits in the dopaminergic responses of EGF model rats to the social stimuli.

### Dual effects of risperidone treatment on social interaction and VTA dopaminergic cell activity

**Fig. 3:**
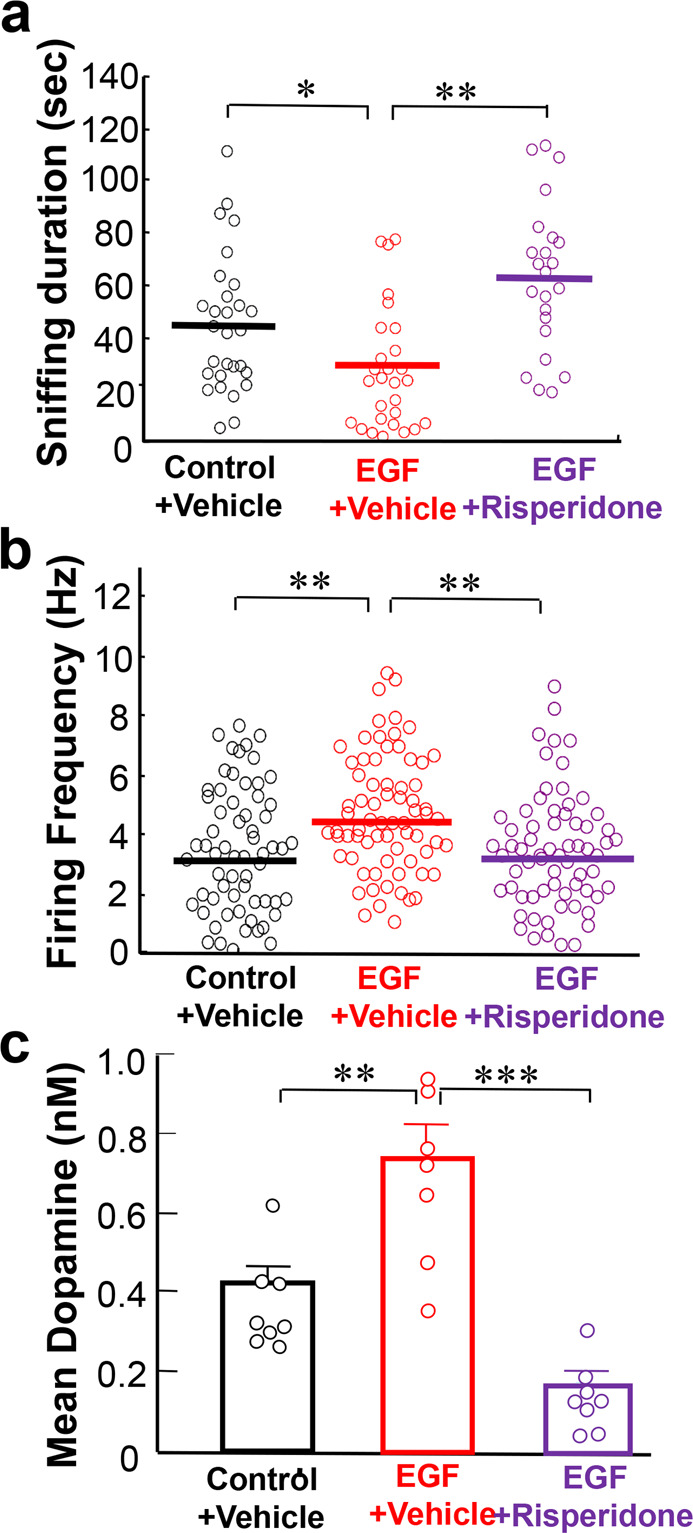
Subchronic antipsychotic actions on social interaction scores, firing activities, and basal dopamine release of epidermal growth factor model rats. **a** Sniffing durations of EGF model rats (*n* = 23) which had subchronically received risperidone (1.0 mg/kg/day, i.p.) were compared with those of EGF model rats (*n* = 28) and control rats (*n* = 29) which had been treated with vehicle by Kruskal–Wallis followed by Steel post hoc test. **b** Following the social interaction test, these rats were anesthetized and subjected to in vivo extracellular single‐unit recordings from putative ventral tegmental area (VTA) dopaminergic neurons. The mean firing frequency of each cell is plotted. (*n* = 63 cells for control + vehicle, *n* = 72 cells for EGF + vehicle, *n* = 67 cells for EGF + risperidone) by Kruskal–Wallis followed by Steel post hoc test. **c** Effects of risperidone on basal dopamine concentrations in the prelimbic cortex of vehicle-treated control rats (*n* = 8), vehicle-treated EGF model rats (*n* = 7), and risperidone-treated EGF model rats (*n* = 8), were determined at the resting state. Prelimbic dopamine at the rat resting state was recovered with the lower flow rate for every 30 min and its concentrations were averaged over the 120 min period. Data were analyzed by one-way ANOVA followed by Tukey post hoc test. *P < 0.05, **P < 0.01, and ***P < 0.001.

We examined the effects of risperidone on social interaction scores and on putative VTA dopaminergic cell firing (Fig. 3). Saline or risperidone (1 mg/kg/day) were intraperitoneally administered daily to EGF model rats and control rats for 2 weeks. In the vehicle-treated group, EGF model rats showed significantly shorter sniffing durations in the social interaction test compared with vehicle-treated control rats (45.1 ± 4.9 s for vehicle-treated control; 28.1 ± 4.6 s for vehicle-treated EGF rats; *T* = 2.67, *P* = 0.015 by Steel post hoc) (Fig. 3a). However, subchronic treatment with risperidone significantly increased the sniffing duration (63.4 ± 6.0 s) in EGF model rats (*T* = 3.91, *P* < 0.001 by Steel post hoc).

The opposite trend to the social interaction scores was observed in the mean firing rates of putative dopaminergic cells in the VTA (Fig. 3b). EGF model rats treated with vehicle exhibited higher firing rates (4.52 ± 0.25 Hz) than vehicle-treated control rats (3.23 ± 0.24 Hz) (*T* = 3.44, *P* = 0.001, by Steel post hoc). Risperidone significantly decreased the firing rates in the EGF model (3.26 ± 0.22 Hz) (*T* = 3.69, *P* < 0.001 by Steel post hoc). In agreement, the baseline dopamine concentrations of these groups of rats showed a similar pattern of differences (*F*_2,20_ = 26.72, *P* < 0.001 by one-way ANOVA, Fig. 3c); dopamine concentrations recovered from the prelimbic cortex were significantly higher in vehicle-treated EGF model rats (0.75 ± 0.08 nM) than in vehicle-treated control rats (0.42 ± 0.04 nM; *P* = 0.0012 by Tukey post hoc). The subchronic risperidone administration normalized the dopamine levels in EGF model rats (0.18 ± 0.03 nM; *P* < 0.001 by Tukey post hoc).

### DREADD-driven suppression of VTA dopaminergic activity ameliorates the social deficits of EGF model rats

**Fig. 4:**
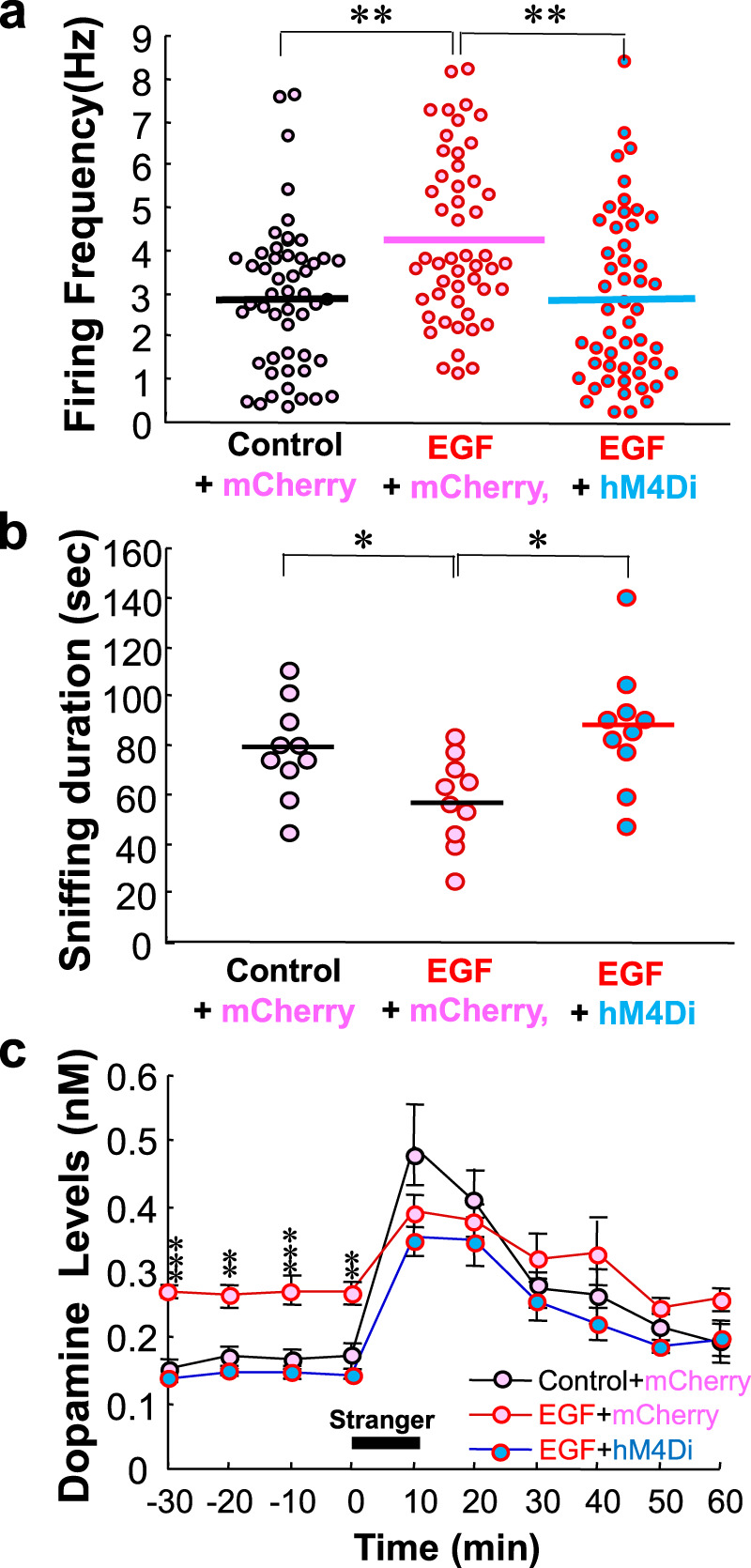
DREADD-driven attenuation of hyperdopaminergic states ameliorates social interaction deficits of epidermal growth factor model rats. **a** AAV vectors carrying the hM4Di gene plus mCherry (hM4Di) or mCherry alone (mCherry) were microinjected into the VTA of EGF model rats or control rats. The hM4Di ligand clozapine N-oxide (CNO) was subchronically administered to these rats with the aid of an osmotic pump. The DREADD effects on firing rates of ventral tegmental area (VTA) dopaminergic neurons were ascertained in EGF model rats carrying the hM4Di gene by comparison with those in EGF model rats or control rats expressing mCherry alone. (*n* = 55 cells, 6 rats for control + mCherry; *n* = 52 cells, 7 rats for EGF + mCherry, *n* = 53 cells, 7 rats for EGF + hM4Di), by Kruskal–Wallis followed by Steel post hoc test. **b** The sniffing duration of these animals were measured as their social interaction scores (*n* = 10 for control rats + mCherry, n = 10 for EGF rats + mCherry, n = 10 for EGF + hM4Di), by Kruskal–Wallis followed by Steel post hoc test. **c** Dopamine release from VTA dopaminergic neurons was monitored in the prelimbic cortex. The baseline levels and social interaction-triggered release of dopamine from the prelimbic cortex were compared among these three groups of rats (*n* = 6 for control + mCherry, *n* = 7 for EGF model rats + mCherry, *n* = 6 for EGF model rats + hM4Di) across 20 bins (1 min each) by two-way repeated ANOVA followed by Tukey post hoc test with the Holm’s compensation. *P < 0.05, **P < 0.01, ***P < 0.001.

To examine the pathological role of the hyperdopaminergic activity in social interaction, we manipulated the VTA dopaminergic cell firing, employing the DREADD approach^41^. The AAV vector carrying the gene for hM4Di receptor or mCherry alone was administered into the VTA of EGF model rats or control littermates of the transgenic rat line expressing *Cre* recombinase in dopaminergic/noradrenergic cells^42^. Our preliminary examination revealed that 48.2 ± 5.9% (*n* = 6) of tyrosine hydroxylase-positive dopaminergic neurons expressed AAV-derived hM4Di in both hemispheres of the VTA of the Cre-driver Long Evans rats (Supplemental Fig. S1). In contrast, the frequency of non-dopaminergic cells expressing hM4Di was very limited (3.9 ± 1.6%), confirming the cell specificity of the present DREADD approach. We subchronically administered the ligand CNO to all groups of rats with the aid of an osmotic pump (600 μg/day/rat) and determined the CNO effects on firing activity of putative VTA dopaminergic neurons under anesthesia (Fig. 4a). In the groups expressing mCherry alone, the firing frequency of the putative dopaminergic cells was 2.94 ± 0.23 Hz in control rats and 4.28 ± 0.26 Hz in EGF model rats, which showed significant difference (*T* = 3.20, *P* = 0.003 by Steel post hoc) as we expected. When the rats carrying hM4Di receptors were treated with CNO, the firing frequency was reduced to 2.88 ± 0.27 Hz (*T* = 3.55, *P* < 0.001, by Steel post hoc), confirming the DREADD effect. With the DREADD-driven suppression of the VTA dopaminergic activity, the sniffing duration of EGF model rats was significantly prolonged (86.7 ± 8.0 s), compared with that of EGF model rats expressing mCherry alone (57.2 ± 5.7 s; *T* = 2.65, *P* = 0.016 by Steel post hoc) (Fig. 4b). The original difference in sniffing duration between the EGF model and control rats both expressing mCherry alone was also reproduced (*T* = 2.26, *P* = 0.043, by Steel post hoc). Thus, the social interaction deficits of EGF model rats were significantly ameliorated by the DREADD-driven attenuation of their higher dopaminergic cell firing at the resting state.

Before and during the social interaction test, we also monitored dopamine release from the prelimbic cortex, one of the target regions of VTA dopaminergic neurons (Fig. 4c). Again, all groups of rats had similarly received CNO from an osmotic pump. Two-way repeated ANOVA with the main factors of EGF and time (i.e., social stimuli) revealed the significant main effects of EGF (*F*_2,16_ = 5.74, *P* = 0.013) and time (*F*_3.84,16_ = 40.8, *P* < 0.001) with an EGF × time interaction (*F*_7.69,16_ = 2.67, *P* = 0.015). The statistical interaction suggested that dopamine concentrations significantly varied during the test period, presumably depended upon the social stimuli. At individual time bin, we further analyzed the rat group difference in dopamine concentrations; Tukey post hoc test with the Holm’s compensation detected significantly lower dopamine concentrations from the M4Di-expressing EGF rats only at the resting state before social stimuli were given (*P* = 1.76 × 10 ^−5^− 0.0018 with Holm’s compensation) but not during or after the social interaction period. The above results of the DREADD manipulation suggest that the higher baseline dopamine release from VTA dopaminergic neurons has a strong impact on the social behaviors of EGF model rats.

### A negative correlation between sniffing duration and baseline dopaminergic cell firing

**Fig. 5:**
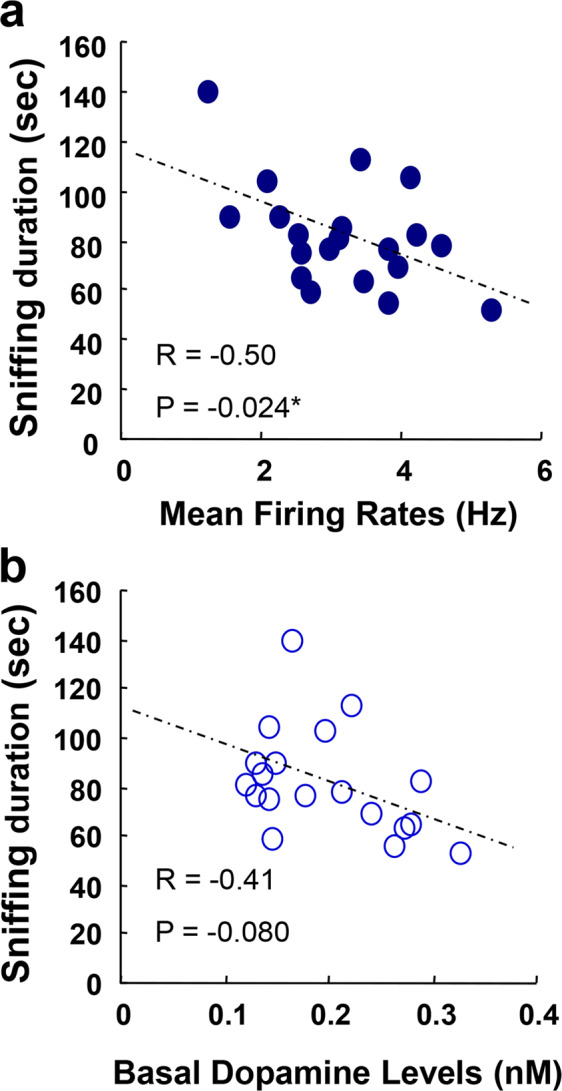
A negative correlation between dopaminergic cell firing in the ventral tegmental area and social interaction score. **a** To evaluate the regulatory strength of the dopaminergic activity on social behaviors in the above DREADD experiment, individual firing rates of the putative VTA dopaminergic neurons in Fig. 4 were averaged for each rat, plotted against their social interaction scores (i.e., their sniffing duration) and subjected to the Pearson’s correlation analysis. **b** Similarly, resting-state dopamine concentrations in the prelimbic cortex were subjected to the correlation analysis with social interaction scores. A rat losing the cannulation probe was excluded from the final analysis. Animal allocation and data sharing between Figs. 4 and 5 are presented in Supplemental Table S1.

To further confirm the above regulatory role of VTA dopaminergic activity, we explored the quantitative correlation between dopaminergic cell firing rate and sniffing duration in the above DREADD experiments. We averaged the firing frequencies of all recorded cells in each rat and allocated their average to each animal (Fig. 5). The Pearson’s correlation analysis revealed a negative correlation between the mean firing rates and sniffing duration of individual rats (*R* = − 0.50, *P* = 0.024; Fig. 5a). In contrast to the correlation of dopaminergic cell firing, terminal concentrations of dopamine in the prelimbic cortex were not significantly correlated with sniffing duration, however (*R* = − 0.41, *P* = 0.080; Fig. 5b).

## Discussion

In the present study, we investigated the neuropathological role of A10 mesocorticolimbic dopaminergic activity in gender-matched affiliative social interactions in the neurodevelopmental model of schizophrenia that is known to exhibit chronically decreased social interactions^35–39^. The main findings of the present study are as follows: (1) Among dopaminergic, noradrenergic, and serotonergic cell firing as monitored under the anesthetic condition, only putative dopaminergic cells of the EGF model exhibited an abnormal increase in their firing frequency but not in their SWB. (2) In the free-moving condition, the baseline firing frequency and SWB of putative dopaminergic cells were elevated in EGF model rats, whereas the social stimuli-triggered firing and bursting were conversely blunted in these rats. The difference of EGF effects on SWB between the presence and absence of anesthesia might suggest an extrinsic effect of afferent nerve activities but not that of intrinsic influences of dopaminergic neurons. (3) Subchronic administration of risperidone to EGF model rats normalized the abnormal firing frequency of putative dopaminergic neurons and the dopamine concentrations at the resting state, concomitant with the amelioration of their social interaction deficit. It is also known that a typical antipsychotic, haloperidol, does not ameliorate the social deficits of this model^35^. Haloperidol treatment of this model fails to alter the dopaminergic cell firing (HS unpublished data). These findings appear to be concordant with the reports that the chronic medication of atypical antipsychotics involving serotonin antagonism is beneficial for treating negative symptoms of schizophrenia patients, although this argument is still controversial^54,55^. (4) DREADD-driven attenuation of baseline VTA dopamine cell firing in EGF model rats restored the event-triggered dopamine release and normalized the social deficits. (5) There was an inverse liner correlation between baseline VTA dopaminergic cell activity and social interaction scores. Although acute dopamine release is known to elevate locomotive behaviors, we did not observe locomotor alterations in the EGF model rats exhibiting chronic hyperdopaminergic activity^35,36,56,57^. With the well-established role of acute phasic dopamine effects on social behaviors, these observations indicate that the baseline activity of the VTA dopaminergic pathway in the resting state has marked negative influences on the following event-triggered dopamine release and its functions. As the dopaminergic neurons in VTA are heterogenous with respect to their connectivity and co-neurotransmitters, it remains to be determined whether the negative effects of the hyperdopaminergic activity in the resting states can be found in all subtypes of VTA dopamine neurons^58,59^.

### Distinct effects of acute and chronic dopamine release on rodent social behaviors

Previous studies explored mainly the regulatory role of the acute phasic dopamine release in rodent social interaction in the mouse models for depression or post-traumatic stress disorder^16,60^. Acute optogenetic manipulation of VTA dopaminergic cell activity is applied to the social interaction behaviors and revealed that local dopamine release from the nucleus accumbens positively regulates social interactions^16,60^. The reports also demonstrated that the local optogenetic stimulation of dopamine release from the prelimbic region failed to influence murine social behaviors. As the present DREADD effects presumably spread over the entire A10 dopaminergic pathway, it is postulated that their dopamine release was elevated not only in the prelimbic cortex but also in the nucleus accumbens of EGF model rats. The significant correlation of social behaviors with VTA dopamine cell firing, but not with prelimbic dopamine concentrations, might propose that dopamine release from the nucleus accumbens plays a more dominant role in the regulation of social behaviors^60^.

Which mechanism was primarily responsible for the regulation of the social interactions in rats; the baseline or event-triggered dopamine release^17,54^? and the tonic or phasic of VTA dopaminergic cell firing^16,60^? A lesion study of A10 dopaminergic fibers and the acute phasic optogenetic activation of VTA dopaminergic neurons both suggest a primary role for acute event-triggered dopamine actions^60–63^. Therefore, the present findings are alternatively explained; the higher baseline activity of VTA dopaminergic neurons resulted in the attenuation of event-triggered dopamine responses that was responsible for the observed social deficits. As the SWB of VTA dopaminergic cells were also elevated in this EGF model, the latter question remains to be addressed with respect to the reported distinct functions of their tonic and phasic firing properties^17,18,30^. Our preliminary study might indicate the limitation of post-synaptic contribution to the above phenomenon; protein concentrations of dopamine receptors D1R and D2R in the prelimbic cortex exhibited no significant difference between groups (Supplemental Fig. S2).

### Minimum side effects of clozapine N-oxide

We previously reported that subchronic treatment with clozapine at a daily dose of 5.0 mg/kg can ameliorate the social interaction deficits in this EGF model. The artificial M3Di receptor of the present DREADD system employed the ligand CNO, which has been suggested to be converted into clozapine that penetrates the brain^64^. It is also reported that the high CNO dose (i.e., 5 mg/kg) exerts behavioral influences of normal mice^65^. Therefore, we carefully controlled the use of CNO in the present study. If all CNO was fully converted into clozapine in vivo, the dose can be calculated as ~1.5 mg/kg/day. Our preliminary study verified that this CNO dose did not influence the social interaction score or locomotive activity of authentic EGF model rats, or the firing frequency of their putative VTA dopaminergic neurons (Supplemental Figs. S3 and S4). Moreover, animals receiving the same CNO dose were used as a control group to evaluate the relative effects of the DREADD-driven dopaminergic manipulation. Based on these considerations, we conclude that unwanted CNO effects were minimized and controlled in the present study, although the present results need to be replicated with other DREADD ligands harboring no affinity to dopamine receptors.

### Dopamine hypothesis and negative symptoms

It is an important question whether the basal hyperdopamine-driven reduction in social behaviors is relevant to the negative symptom of schizophrenia^66^. Okubo et al.^67^ reported that the negative symptom of schizophrenia is associated with the decreased availability of prefrontal D1 receptors. Lehrer et al.^68^ suggested the reduced availability of prefrontal dopamine D2 receptors in patients with schizophrenia. Although it is unknown whether these decreases in dopamine receptor occupancy represent a sustained increase in dopamine release or the up-regulation of dopamine receptors^17–22^, several studies using functional magnetic resonance support the former possibility^69,70^.

Previous studies on drug addiction had provided rebutting evidence that chronic amphetamine intake fails to induce social withdrawal or other negative symptoms of schizophrenia^71,72^. Rather, the abuse of phencyclidine or *N*-methyl-D-aspartate (NMDA) receptor antagonists have been proposed to induce negative symptoms in humans, and their chronic administration or NMDA receptor ablation results in negative symptom-like behaviors in rodents^73–77^. However, the reports that both chronic phencyclidine administration and NMDA receptor blockade persistently potentiate or evoke dopamine release might warrant the revision of this argument^78–81^.

### Dopamine release abnormality in this model and patients with schizophrenia

Positron emission tomography studies on schizophrenia suggest that the hypodopaminergic state in the prefrontal cortex and the hyperdopaminergic state in the striatum are associated with this disorder^81^, although controversies and heterogeneity among patients still remain^82,83^. Although the term “hypodopaminergic” or “hyperdopaminergic” is confusing unless the proper comparison target is specified. In general, these terms are defined as the degree of event/test-evoked dopamine responses but not as baseline dopamine release at the resting state^17,31^. Thus, the previous findings that the task/drug-triggered dopamine release is blunted in the prefrontal cortex of patients with schizophrenia^19,84,85^ appear to match the present dopamine dynamics of EGF model rats; the event-triggered changes in VTA dopamine activity were attenuated during the social interaction period. There were a few reports implying increases in the basal dopamine release or the resting-state activity of the A10 dopamine system, however^70,71^. Rodent social interactions comprise very complex behaviors involving not only attention and anxiety, but also olfactory cognition and memory ability^72^. At present, therefore, it is unknown whether the observed deficits in the present social interaction paradigm reflected the behavioral impairments stemming from the dopamine-driven cognitive domain or social domain.

## Supplementary information

**Figure S1**; Estimation of hM4Gi/mCheery-positive cell ratios in VTA. **Figure S2**; Dopamine receptor-like immunoreactivities in the prelimbic cortex of control and EGF model rats. **Figure S3**; Evaluation of CNO effects on social behaviors and firing rates of putative dopaminergic cells in EGF-challenged Long-Evans rats. **Figure S4**; Influences of CNO on exploratory locomotion of EGF-challenged Long-Evans rats. **Table S1**; Animal allocation and data correspondence. **Table S2**; Statistical data and tests applied.
