# Supplementary material for: Resting-state dopaminergic cell firing in the ventral tegmental area negatively regulates affiliative social interactions in a developmental animal model of schizophrenia
Source: Transl Psychiatry. 2021 Apr 22;11:236. doi: 10.1038/s41398-021-01346-2 (PMC8062445; doi:10.1038/s41398-021-01346-2)
Supplement: Supplementary file 1 — Figure S1; Estimation of hM4Gi/mCheery-positive cell ratios in VTA. Figure S2; Dopamine receptor-like immunoreactivities in the prelimbic cortex of control and EGF model rats. Figure S3; Evaluation of CNO effects on social behaviors and firing rates of putative dopaminergic cells in EGF-challenged Long-Evans rats. Figure S4; Influences of CNO on exploratory locomotion of EGF-challenged Long-Evans rats. Table S1; Animal allocation and data correspondence. Table S2; Statistical data and tests applied. [file 41398_2021_1346_MOESM1_ESM.pdf]

SUPPLEMENTARY Materials

Resting state dopaminergic cell firing in the ventral tegmental area negatively regulates affiliative social interactions in a developmental animal model of schizophrenia

H. Sotoyama, H. Namba, Y. Kobayashi, M. T Hasegawa, D. Watanabe, E. Nakatsukasa, K. Sakimura, T. Furuyashiki, and H. Nawa

FIGURE S1; Estimation of hM4Gi/mCheery-positive cell ratios in VTA.

FIGURE S2; Dopamine receptor-like immunoreactivities in the prelimbic cortex of control and EGF model rats.

FIGURE S3 ; Evaluation of CNO effects on social behaviors and firing rates of putative dopaminergic cells in EGF-challenged Long-Evans rats.

FIGURE S4; Influences of CNO on exploratory locomotion of EGF-challenged Long-Evans rats.

Table S1; Animal allocation and data correspondence.

Table S2; Statistical data and tests applied.

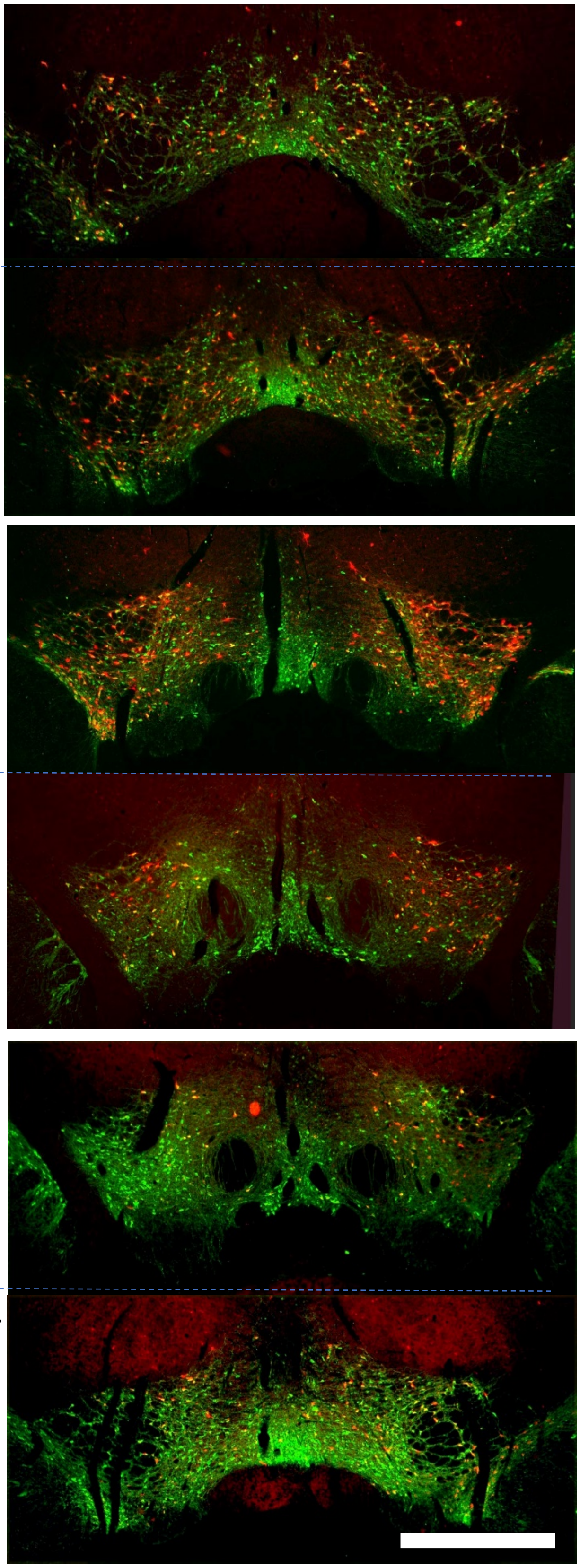

TH-cre Rat1

Cell ratio of mCherry+TH+/TH+
= 0.435
Cell ratio of
mCherry alone +/mCherry+
= 0.000

Cell ratio of mCherry+TH+/TH+
= 0.494
Cell ratio of
mCherry alone +/mCherry+
= 0.026

TH-cre Rat2

Cell ratio of mCherry+/TH+
= 0.627
Cell ratio of
mCherry alone +/mCherry+
= 0.024

Cell ratio of mCherry+TH+/TH+
= 0.664
Cell ratio of
mCherry alone +/mCherry+
= 0.028

TH-cre Rat3

Cell ratio of mCherry+TH+/TH+
= 0.295
Cell ratio of
mCherry alone +/mCherry+
= 0.112

Cell ratio of mCherry+TH+/TH+
= 0.376
Cell ratio of
mCherry alone +/mCherry+
= 0.042

Summary

Cell ratio of mCherry+TH+/TH+
= 0.482+/-0.059
Cell ratio of mCherry alone +/mCherry+ = 0.039+/-0.016

Supplemental Figure S1; Estimation of hM4Gi/mCheery-positive cell ratios in VTA. AAV vectors was injected into the left and right hemispheres of VTA of the Cre-driver rats (N=3 x 2). The central areas of the VTA were immunostained with the anti- tyrosine hydroxylase (TH) antibody (Green). Note: The colour balance between red and green channels was optimized to count their double staining. The anti-Th antibody was obtained from Chemicon Int (1: 300 dilution; Catalogue#152). A white bar represents 2.0 mm.

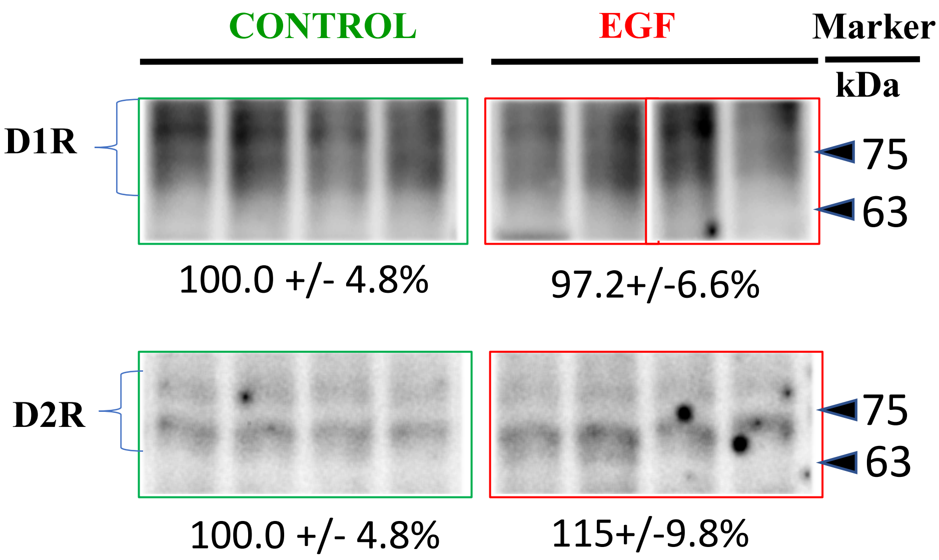

Supplemental Figure S2; Dopamine receptor-like immunoreactivities in the prelimbic cortex of control and EGF model rats. After behavioral tests, EGF-challenged rats were dissected and the tissue of their prelimbic cortex was taken and stored in a deep freezer. The tissue homogenates were subjected to immunoblotting with the knockout mouse-validated antibodies (Nittobo Medical Ltd., Tokyo Japan) raised against dopamine receptors D1R (https://nittobo-nmd.co.jp/pdf/reagents/D1R.pdf) and D2R (https://nittobo-nmd.co.jp/pdf/reagents/D2R.pdf). The immunoreactivities for the dopamine receptors are known to be distributed broadly with the given glycosylation and sensitive to heat-denaturation. There were no significant differences in the total strength of their immunoreactivity between groups.

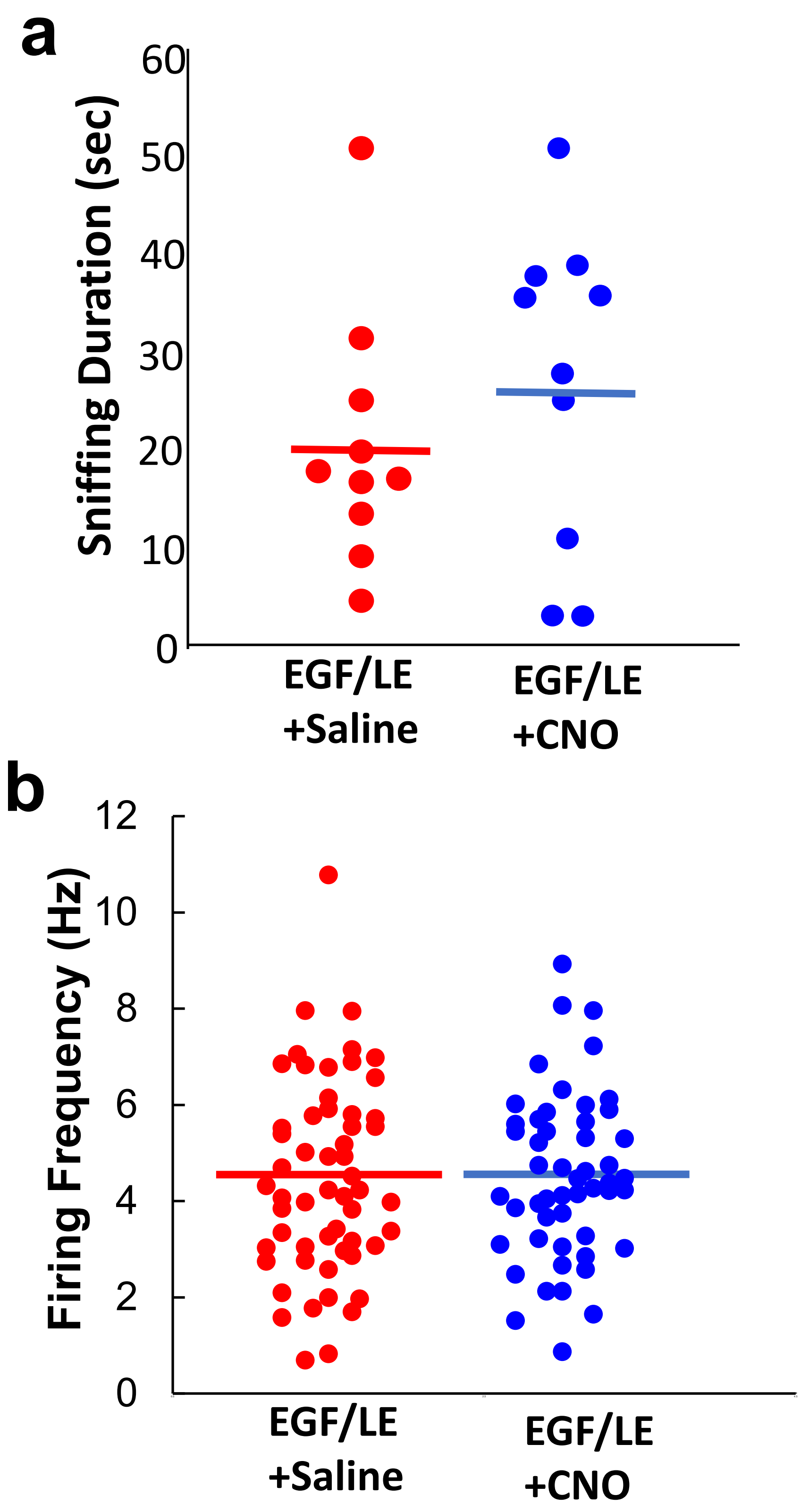

Supplemental Figure S3; Evaluation of CNO effects on social behaviors and firing rates of putative dopaminergic cells in EGF-challenged Long-Evans rats. CNO or saline were subchronically administered from osmotic pumps to EGF-challenged rats without AAV infection (N=10 rats each). The dose of CNO (~1.5mg/kg/day) had no significant influences on sniffing duration (a) or on firing frequency of putative dopaminergic cells in VTA (b). Horizontal bars represent means.

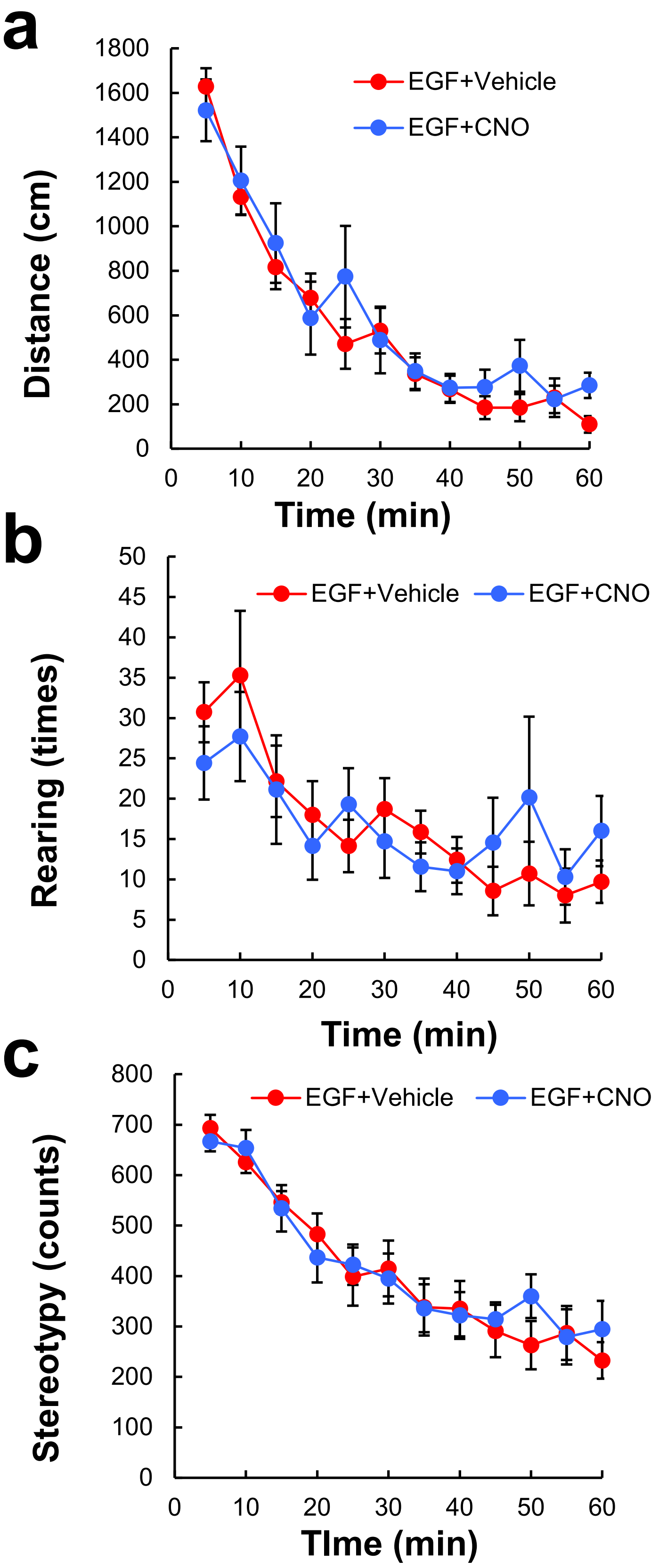

Supplemental Figure S4; Influences of CNO on exploratory locomotion of EGF-challenged Long-Evans rats. CNO or saline was subchronically administered from an osmotic pump to the EGF-challenged rat without AAV infection (N=7 rats each). The dose of CNO (~1.5mg/kg/day) had no significant effects on horizontal movement (a; Distance), rearing movement (b; Rearing), and repeated movement (c; Stereotypy).

Supplemental Table S1; Animal Allocation and Data Correspondence between Figure 4 and Figure 5.

Using the data of Figure 4, we performed Pearson's correlation analysis. Each animal carrying mCherry or hM4Di viral vector and treated with CNO was subjected to the experimental sequence of social interaction test (sniffing), in vivo dialysis of dopamine (DA), and VTA unit recording as shown below. Some of the rats were fixed with paraformaldehyde solution without the experiments of in vivo dialysis and VTA unit recording. The rat (animal #27) which broke the guide cannula on the head was excluded from the in vivo dialysis experiment.

Animal	Group	VIRUS	CNO DREADD	Sniffing (sec) **Fig4b & Fig5ab**	Basal DA (nM) **Fig4c & Fig5b**	mean VTA firing (Hz) **Fig4a & Fig5a**	Other Experimental Use
1	Control	mCherry	+	75.59	0.142779147	2.566666667	
2	Control	mCherry	+	80.96	0.122210427	3.11125	
3	Control	mCherry	+	103.29	0.19715163	2.108181818	
4	Control	mCherry	+	76.36	0.179172559	3.812	
5	Control	mCherry	+	112.63	0.220888045	3.409230769	
6	Control	mCherry	+	89.73	0.129941873	2.26	
7	Control	mCherry	+	71.63			histchemistry
8	Control	mCherry	+	58.47			histchemistry
9	Control	mCherry	+	44.76			histchemistry
10	Control	mCherry	+	80.53			histchemistry
11	EGF	mCherry	+	65.13	0.278057305	2.556	
12	EGF	mCherry	+	52.29	0.325807771	5.298461538	
13	EGF	mCherry	+	69.51	0.240656875	3.945	
14	EGF	mCherry	+	63.09	0.272071881	3.474	
15	EGF	mCherry	+	55.31	0.262371643	3.803333333	
16	EGF	mCherry	+	82.64	0.288317193	4.212727273	
17	EGF	mCherry	+	77.43	0.212562238	4.557272727	
18	EGF	mCherry	+	38.56			histchemistry
19	EGF	mCherry	+	23.98			histchemistry
20	EGF	mCherry	+	43.97			histchemistry
21	EGF	hM4Di	+	139.89	0.164236006	1.23	
22	EGF	hM4Di	+	90.03	0.150310211	1.5775	
23	EGF	hM4Di	+	59.06	0.145744032	2.693333333	
24	EGF	hM4Di	+	77.01	0.130006427	2.989	
25	EGF	hM4Di	+	104.68	0.14388087	4.12625	
26	EGF	hM4Di	+	84.77	0.137679069	3.153333333	
27	EGF	hM4Di	+	82.43	gui de di sconnect	2.522666667	
28	EGF	hM4Di	+	92.84			histchemistry
29	EGF	hM4Di	+	46.16			histchemistry
30	EGF	hM4Di	+	89.63			histchemistry

Supplemental Table S2; Statistical data and tests applied. In order to select proper statistical test, all data were initially subjected to the Kolmogorov–Smirnov or Shapiro-Wilk test for the normal distribution and the M or Leveve test for homogeneity of variances, followed by the parametric or nonparametric comparisons. To keep the statistical power, the same statistical comparisons were applied in the same experimental category of data to keep the statistical power in the same experiment.

Fig	explanatory variable	dependent variable	sample size	distribution normality	variation homogeneity	statistical test	comparisons	statistical values	probability	Multiple Comparison Compensation
				Smirnov or Shapiro-Wilk	Box's M or Levene	Main post-hoc				Holm etc
			48/46	0.346, 0.986	0.516	Mann-Whitney	CON vs EGF	U=810.5, Z=2.22	0.026	
1A-1C	CON/EGF	frequency	42/41	0.119, 0.013	0.304	Mann-Whitney	CON vs EGF	U=805.5, Z=0.506	0.506	
			44/41	0.600, 0.051	0.166	Mann-Whitney	CON vs EGF	U=866.5, Z=0.312	0.755	
2A	CON/EGF	sniffing duration	8/8	0.011, 0.838	0.827	Mann-Whitney	CON vs EGF	U=9, Z=2.416	0.016	
	CON/EGF	frequency before	20/17	0.920, 0.045	0.002	Mann-Whitney	CON vs EGF	U=82, Z=2.681	0.007	0.021
		frequency after	20/17	<0.001, <0.001	0.128	Mann-Whitney	CON vs EGF	U=115, Z=1.676	0.094	0.188
2B	before/after	frequency CON	20/20	0.920, <0.001	0.690	Mann-Whitney	Before vs After	U=104, Z=2.599	0.009	0.027
		frequency EGF	17/17	0.045, <0.001	0.244	Mann-Whitney	Before vs After	U=123, Z=0.741	0.459	0.459
	CON/EGF, time	frequency in each time	20/17, (20)	<0.001-0.862	0.035-0.704	Mann-Whitney	CON vs EGF	U=77.5-147, Z=0.701-2.82	0.008-0.483	NA
		mean SWB before	20/17	0.286, 0.640	0.161			main before F=10.06	0.002	
	CON/EGF vs	mean SWB after	20/17	0.560, 0.344	0.787	two-way	CON/EGF vs	main EGF F=6.113	0.0158	
	before/after	mean SWB CON	20/20	0.286, 0.560	0.545	ANOVA	before/after	interaction F=0.909	0.343	
2C		mean SWB EGF	17/17	0.640, 0.344	0.660					
						Tukey	each pairs		0.0239	done
	CON/EGF, time	SWB in each time	18/12, (20)	<0.001-0.862	0.035-0.704	Mann-Whitney	CON vs EGF	U=83-147, Z=2.652-0.701	0.008-0.483	NA
3A	CON/EGF/RIS	sniffing duration	29/28/23	0.048-0.943	<0.001	Kruskal-Wallis	3 goups	H=17.97	<0.001	
						Steel	2 pairs	T=3.91, 2.67	0.001, 0.0147	done
3B	CON/EGF/RIS	frequency	63/72/67	0.001-0.042	0.479	Kruskal-Wallis	3 goups	H=17.27	<0.001	
						Steel	2 pairs	T=3.69, 3.44	<0.001, 0.001	done
3C	CON/EGF/RIS	DA level	8/7/8	0.145-0.919	0.053	one-way ANOVA	3 goups	F(2,20)=26.72	<0.001	
						Tukey	each pairs		<0.001-0.012	done
	CON+mCherry/					Kruskal-Wallis	3 goups	H=15.20	<0.001	
4A	EGF+mCherry/	frequency	55/52/53	0.003-0.139	<0.001					
	EGF+M4Di					Steel	2 pairs	T=3.20, 3.55	<0.001, 0.003	done
	CON+mCherry/					Kruskal-Wallis	3 goups	H=8.47	0.0144	
4B	EGF+mCherry/	sniffing duration	10/10/10	0.286-0.916	0.93					
	EGF+M4Di					Steel	2 pairs	T=2.65, 2.27	0.016, 0.044	done
	CON+mCherry/					two-way	3 goups	main EGF F=5.741	0.013	
	EGF+mCherry/	DA level for all	6/7/6 x 20	0.063-0.966	0.052-0.689	repeated ANOVA		main time F=40.80#	<0.001	
	EGF+M4Di, time						each time	interaction F=2.668#	0.015	
4C	CON+mCherry/					one-way ANOVA	3 goups	F=0.56-18.0	<0.001-0.58	
	EGF+mCherry/	DA level in each time	6/7/6	0.063-0.966	0.0524-0.640					
	EGF+M4Di					Tukey	each pairs at each time bin		<0.001-0.843	<0.001-1.0
5A	sniffing		20	0.101		Peason		T=2.5	0.024	
	DA frequency		20	0.974						
5B	sniffing		19	0.186		Peason		T=0.39	0.08	
	DA level		19	0.048						
Supple S3A	EGF+Saline/	sniffing duration	10/10	0.123, 0.340		Student T	saline vs CNO	T=0.958	0.351	
	EGF+CNO				0.543					
Supple S3B	EGF+Saline/	frequency	56/51	0.562, 0.737	0.246	Student T	saline vs CNO	T=0.0560	0.955	
	EGF+CNO									
						two-way *	saline vs CNO	main EGF F=0.253	0.624	
Supple S4A	EGF+Saline/	Distance	7/7 x 12	0.138-0.964	0.005(a)-0.876	repeated ANOVA		main time F=63.971#	<0.001	
	EGF+CNO, time							interaction F=1.261#	0.254	
					a)0.005	Mann-Whitney	saline vs CNO	U=24, Z=0.064	0.949	done
						two-way *		main EGF F=0.000	0.990	
				0.017(b-e)-0.954	0.177-0.925	repeated ANOVA	saline vs CNO	main time F=6.914#	<0.001	
	EGF+Saline/							interaction F=1.207#	0.321	
Supple S4B	EGF+CNO, time	Rearing	7/7 x 12	b)0.039		Mann-Whitney	saline vs CNO	U=18.5, Z=0.768	0.442	1.000
				c)0.028		Mann-Whitney	saline vs CNO	U=20, Z=0.575	0.565	1.000
				d)0.019		Mann-Whitney	saline vs CNO	U=24.5, Z=1.282	0.200	0.800
				e)0.017		Mann-Whitney	saline vs CNO	U=20, Z=0.575	0.565	0.565
						two-way *		main EGF F=0.0370	0.850	
Supple S4C	EGF+Saline	Stereotpy	7/7 x 12	0.028(f)-0.938	0.068-1.000	repeated ANOVA	saline vs CNO	main time F=37.73#	<0.001	
	EGF+CNO							interaction F=0.778#	0.559	
				f)0.028		Mann-Whitney	saline vs CNO	U=21, Z=0.447	0.655	done

Abbreviations used>>>CON; control rats, EGF;EGF-challenged rats, DA; dopamine levels, SWB; spikes within bursts

Note 1; NA denotes Not Applied with the given high number of groups

Note 2; # denotes the Greenhouse-Geisser compensation.

Note 3; * Rat locomotion scores are known to be destibuted with the Gaussian distribution.

Note 4; a-f) represent the expeptional data points violating the normality or homogeniety and thus are subjected to Mann-Whittey test additonally.
